# Using Ecological Momentary Assessment to Investigate Short-Term Variations in Sexual Functioning in a Sample of Peri-Menopausal Women from Iran

**DOI:** 10.1371/journal.pone.0117299

**Published:** 2015-02-18

**Authors:** Amir H. Pakpour, Mir Saeed Yekaninejad, Gianandrea Pallich, Andrea Burri

**Affiliations:** 1 Social Determinants of Health Research Center, Qazvin University of Medical Sciences, Qazvin, Iran; 2 Department of Epidemiology and Biostatistics, School of Public Health, Tehran University of Medical Sciences, Tehran, Iran; 3 Department of Psychology, University of Zurich, Zurich, Switzerland; Catholic University of Sacred Heart of Rome, ITALY

## Abstract

The investigation of short-term changes in female sexual functioning has received little attention so far. The aims of the study were to gain empirical knowledge on within-subject and within- and across-variable fluctuations in women’s sexual functioning over time. More specifically, to investigate the stability of women´s self-reported sexual functioning and the moderating effects of contextual and interpersonal factors. A convenience sample of 206 women, recruited across eight Health care Clinics in Rasht, Iran. Ecological momentary assessment was used to examine fluctuations of sexual functioning over a six week period. A shortened version of the Female Sexual Function Index (FSFI) was applied to assess sexual functioning. Self-constructed questions were included to assess relationship satisfaction, partner’s sexual performance and stress levels. Mixed linear two-level model analyses revealed a link between orgasm and relationship satisfaction (Beta = 0.125, P = 0.074) with this link varying significantly between women. Analyses further revealed a significant negative association between stress and all six domains of women’s sexual functioning. Women not only reported differing levels of stress over the course of the assessment period, but further differed from each other in how much stress they experienced and how much this influenced their sexual response. Orgasm and sexual satisfaction were both significantly associated with all other domains of sexual function (P<0.001). And finally, a link between partner performance and all domains of women`s sexual functioning (P<0.001) could be detected. Except for lubrication (P = 0.717), relationship satisfaction had a significant effect on all domains of the sexual response (P<0.001). Overall, our findings support the new group of criteria introduced in the DSM-5, called “associated features” such as partner factors and relationship factors. Consideration of these criteria is important and necessary for clinicians when diagnosing FSD.

## Introduction

Female sexual dysfunction (FSD) describes a range of disorders related to sexual desire, arousal, orgasm, and sexual pain [1]. Although today sexual problems are commonly accepted to be a multifactorial phenomenon, involving—among other—anatomical, physiological, biological, and psychological factors, more recently, research emphasis has been placed on environmental and interpersonal factors, such as the role of stress, relationship quality, and partner performance for a woman’s sexual functioning [2–6].

Numerous studies have suggested that greater feelings of love, lower levels of marital conflict, and greater marital happiness are related with satisfying sexual relationships [7–9]. These results provide the conceptual basis for the hypothesis that relationship satisfaction plays an important role in women’s sexual functioning and is associated with fewer sexual problems and higher sexual satisfaction [10–12].

Stress is another recently discussed factor in the pathogenesis of sexual problems—although studies have been few-, describing a complex of outside pressures which bear down on mental and physical well-being e.g., [13]. Nowadays, women have to deal with an expanding complex of pressures while trying to balance work, family life, and marriage. External stressors and daily hassles (in other words, events that are not always controllable) sometimes become so overwhelming that they not only impact on general well-being but consequently also on sexual relation and functioning [14]. For some women stress can lead to a decrease in sexual desire or interest in engaging in sex, for others, sex might even become an additional stressor in their lives, making it impossible for them to experience an enjoyable sexual interaction with their partner [15–16].

Partner’s sexual functioning is also likely to affect female sexual functioning. A wealth of studies has provided consistent evidence for the impairing effects of male partner’s sexual problems for women’s sexuality. Female partners of men with erectile dysfunction (ED) or premature ejaculation (PE), for example, have been shown to report less sexual satisfaction and more sexual problems compared to female partners of healthy men [17–18].

Despite solid evidence for the important role of stress, relationship imbalances and partner performance in the etiology of women’s sexual problems, to date and to the best of our knowledge, no studies have investigated women’s sensitivity to the short-term influences of these factors. Furthermore, there is a lack of knowledge regarding the stability and/or variability of the various domains of female sexual functioning across time. But certain definitions and diagnostic criteria (e.g. “persistent” in the DSM-5) imply temporal stability and continuity, and therefore a more in depth knowledge about variability and temporal stability of sexual functioning is needed in order to promote the formulation of more accurate clinical definitions and diagnostic criteria for FSD [1].

Overall, more accurate clinical definitions—particularly in terms of onset of symptoms, duration, cause and context—can provide useful information and insights into the nature and etiology of the disorder, particularly when it is acquired or situational e.g., [1,19]. For each diagnosis it is therefore imperative to determine the length of time the disorder has existed (lifelong vs. acquired), the extent to which it is partner- or situation-specific as opposed to occurring in all situations (generalized vs. situational), the degree of distress, and whether it is due to psychological, physiological or a combination of factors [1,20–21]. A study conducted on 1489 British women, for example, recently demonstrated how the etiologic structures underlying short-term (i.e. past 4 weeks) versus long-term (i.e. lifelong) sexual functioning differ from each other by suggesting a more accentuated influence of biologic factors on enduring patterns of sexual functioning, as opposed to etiological factors which seem to have a stronger impact on short-term fluctuations in sexual functioning [22]. Given this discordance, it is important to understand which factors contribute to innate inter-individual differences and which factors provoke phasic, intra-individual changes in sexual functioning, and how differential psychosocial experiences may account for such short-term changes. Equally important is the knowledge on how sexual experiences in an individual may influence or even predict subsequent levels of sexual function.

However, most of the epidemiological studies on FSD use a cross-sectional design, therefore allowing assessment of sexual function at one specific point of time only. In other words, the stability and variability of women’s sexual function levels and the factors that causes them to alter remain largely unknown. To close this gap, the present study proposes a “short-term” longitudinal approach that allows a deeper understanding of the progress of these variables and what may cause them to alter. By using ecological momentary assessment (EMA), repeated sampling of real time data on subjects’ current behavior and experience in their natural environments is possible [23]. This strong design captures “day-to-day” sexual activities” and consequently allows data assessment in a natural context, by simultaneously minimizing cognitive distortion or recall bias.

## Aims

The aims of this explorative study were two-fold. 1. To assess intra-individual variation across various domains of sexual functioning in a population sample of Iranian women. More specifically, to investigate the stability of self-reported sexual functioning (i.e. intra-individual differences) across sexual activities on all subdomains, and to describe the changes of sexual functioning between the women (i.e., inter-individual differences); 2. To explore the effects of stress, relationship satisfaction and partner performance on women’s sexual functioning.

## Materials and Methods

### Sample and Recruitment

The present study was approved by the Ethics Committee of the Qazvin University of Medical Sciences and conducted in a city in Northern Iran. Study participants were a convenience sample of 206 women recruited from a total of eight Urban Health Centers across the city of Rasht. The sample was initially recruited to participate in another project related to menopausal transition, which explains the relatively high mean age of participants. Data assessment took place between March and June 2012. The following inclusion criteria were applied: experiencing natural menopause for at least one year; the ability to read and write Persian; being married or having a steady partner (i.e., being in a relationship for at least 6 months); being in a heterosexual relationship; having an active sex life. Women were excluded if they were: undergoing hormone replacement therapy (HRT); suffering from cognitive impairment (assessed by the midwife with the Mini-Mental State Examination using a cut-off score of < 25); suffering from any systemic or chronic disease, which may interfere with sexual function (such as diabetes, cancer, chronic kidney disease, cardiovascular diseases). From 386 women expressing their interest in participating in the present study, a total of 206 individuals met the inclusion criteria.

Interested participants were invited to come for an informational meeting with the head midwife of the clinic. At that meeting, the study was described in detail and participants were able to ask questions about the study. After providing written consent, the participants were handed out the EMA event log and were carefully instructed on how to fill in the instrument. The EMA event log was provided in a paper version and participants were asked to either send the questionnaire back in pre-paid envelopes or to hand them in at their next visit to the Clinic. In conformity with the event log design, women were instructed to complete the questionnaire as soon as possible after each sexual activity (event log) over a period of six weeks. On days, where no sexual activities took place, women did not have to fill in the questionnaire. Women were further advised to only report on sexual activities involving sexual intercourse and not on other, non-penetrative activities (e.g., petting).

### Instruments

Socio-demographic information on all participants was obtained through available patient data from the clinics. The EMA event log consisted of 20 items of which 16 focused on women’s sexual functioning. The items assessing levels of desire (2 items), arousal (3 items), lubrication (3 items), orgasm (3 items), pain (2 items) and sexual satisfaction (3 items) were taken from the Female Sexual Function Index (FSFI) [24]. The FSFI is a multidimensional self-report instrument for the assessment of female sexual function during the past 4 weeks [9]. The questionnaire can be administered to women across a wide age range, including peri- and post-menopausal women. To prevent missing data (e.g. from non-response) or drop-outs we used a shortened version of the FSFI, so that not all items for each scale were included in the event log. Altogether, 3 items were dropped from the original questionnaire and chosen according to their factor load and relevance (as described in the initial validation study conducted by Rosen et al. 2000) [24]. These items included an item on arousal: “Over the past 4 weeks, how often did you feel sexually aroused ("turned on") during sexual activity or intercourse?” (factor load 0.63); one item on lubrication: “Over the past 4 weeks, how difficult was it to become lubricated ("wet") during sexual activity or intercourse? (factor load 0.74); and one item assessing sexual pain: “Over the past 4 weeks, how often did you experience discomfort or pain during vaginal penetration?” (factor load 0.83). The response options ranged from zero to five, with higher scores indicating better sexual functioning. The initial and following FSFI validation studies showed a high degree of internal consistency (Cronbach`s α values of 0.82 and higher), and excellent psychometric properties, including good reliability, high test re-test reliability for each domain (r = 0.79 to 0.86) and good construct validity.

In a very recent validation study on a sample of 448 Iranian women, the culturally adapted Iranian version of the questionnaire (IV-FSFI) found high reliability coefficients (r ranging from 0.73 to 0.86) and acceptable internal consistencies (Cronbach`s α values ranging from 0.72 to 0.90) [25]. Principal component analysis further confirmed the domain structure, supporting the factorial validity of the IV-FSFI. Recently, a brief 6-item version of the FSFI has been designed and provided further evidence for its validity, also for this shortened version [26].

Further included in the EMA questionnaire were two study-specific items assessing the partner’s sexual function (“Did your partner experience difficulty in getting an erection?” and “Did your partner experience difficulty in maintaining an erection?”), one item assessing relationship satisfaction immediately before engaging in sexual activity (“How would you rate the relationship satisfaction immediately before the sexual activity?”) and one item assessing the degree of self-perceived stress immediately before the sexual encounter (“In general, how stressed were you immediately before the sexual activity?”). These questions were self-constructed with response options ranging from 1 (not at all/very low) to 5 (always/very high).

### Ecological Momentary Assessment (EMA)

EMA is frequently used to study a wide range of behaviors, experiences and symptoms within clinical disorders, as well as life style behaviors such as smoking [27,28]. It permits examination of fluctuations of phenomena over time and about the interactions among these factors by minimizing cognitive distortion or recall bias [23]. Because data is repeatedly collected close in time to the experience and in the women’s natural environment, the ecological validity of this approach is very satisfactory [23].

Retrospective self-reports of various states tend to limit the accuracy of interpretations and reconstructions of a subject’s behavior in the real world setting, as it misses the crucial element of the day to day dynamics [29]. Capturing the progresses of women‘s current state in various live situations, as well as their feelings and reactions in their true disposition in a range of daily situations is particularly crucial in sex research, since the nature of sexual behavior is more conveyed on a daily basis and in real world settings rather than in a controlled setting, such as a laboratory.

### Statistical analyses

Sample characteristics for socio-demographic and sexuality-related variables were reported on the basis of means and standard deviations or numbers and percentages, as appropriate. Univariate and multivariate normality of the various subscales was assessed by visual examination and with Kolmogorov–Smirnov test. All domains showed considerable skewness and kurtosis but were normally distributed after being either log, square or square root transformed.

One way of examining stability across the six weeks in sexual functioning is by estimating an unconditional random intercept two-level model. Between-person and within-person variation in self-reported sexual functioning was examined using two level linear mixed models where repeated time measures were units of the first level (i.e. six assessment points) and women were in second level [30,31]. For estimating the age adjusted effect of orgasm, sexual satisfaction and partner performance (ED) on relationship satisfaction, separate two level random coefficient models were fitted with each of those variables and age as independent variables and with two random effects, one random effect for intercept and another one for the effect of that variable on relationship satisfaction. To find the effect of orgasm, sexual satisfaction, partner performance (ED), relationship satisfaction and stress on domains of women sexual function, multivariate multilevel models were conducted with domains of sexual functioning as response variables and each of the above mentioned variables and age as independent variables. Due to the low numbers of women reporting more than six episodes of sexual intercourse (maximum of reported episodes being 11), subsequent assessment points were not included in the multivariate analyses to avoid the convergence problem in numerical iterative methods

Data cleaning and descriptive analyses were conducted using STATA software (StataCorp., College Station, TX). MLwin statistical software package was used for multilevel modeling using maximum likelihood estimation. In all models, Wald tests were used for testing the hypotheses and calculating the p values. For all analyses, a P value less than 0.05 and 95% confidence interval (CI) were considered statistically significant, unless stated otherwise. The power analysis conducted with G*Power 3.1 showed that statistical tests used for evaluation of our hypotheses given the sample size had an acceptable statistical power of 85%.

## Results

### Sample Description

Because of the specific sample profile, mean age of participants was relatively high (56 years; see Table 1). Similarly, marriage duration was relatively long with a mean of 34 years. All but one participant reported having children. The majority of women reported having sex once or twice times a month (39%), whereas around every fourth woman reported engaging in sexual activities more than once a week (23%).

**Table 1:** Sample characteristics (n = 206).

**Variable**	**Mean**	**SD**	**95% CI**
Age (years)	55.78	6.33	54.92–56.64
Years of marriage	33.86	6.84	32.93–34.79
BMI	27.55	4.35	26.96–28.14
Desire	2.29	0.96	2.16–2.42
Arousal	2.04	1.02	1.9–2.18
Lubrication	3.13	0.64	3.04–3.22
Pain	2.69	1.02	2.55–2.83
Orgasm	2.36	0.63	2.27–2.45
Sexual satisfaction	2.29	0.92	2.16–2.42
Partner’s sexual functioning	2.10	0.96	1.97–2.23
Relationship satisfaction	3.17	1.01	3.03–3.31
Stress	1.91	0.92	1.78–2.04
	N	%	
Education			
*Illiterate*	62	30.1	
*Primary school*	54	26.2	
*Secondary school*	41	19.9	
*Diploma*	44	21.4	
*Associate Degree*	5	2.4	
*BA and upper*	0	0	
Children, yes	205	99.5	
Disease, yes	110	53.4	
Sexual activity			
*Less than once a month*	66	32.0	
1–2x a month	81	39.3	
*Once a week*	48	23.3	
*3–4x a week*	9	4.4	
*More than 4x a week*	2	1.0	

Note: Desire, arousal, orgasm, relationship and sexual satisfaction are inversely scored, i.e. the higher the score, the fewer problems are reported.

Descriptive statistics of the items measuring women’s sexual functioning, partner sexual performance, relationship satisfaction and stress levels can be found in Table 1 (item means for each variable across all assessment points). On average, participants reported low stress levels and high relationship satisfaction. In terms of sexual functioning, the most frequently reported sexual problem was low arousal and low lubrication (Table 1; note that desire, arousal, orgasm, relationship and sexual satisfaction are inversely scored, i.e. the higher the score the less problems are reported on the specific domain) whereas anorgasmia was the least frequent problem.

### Relationship Between the Various Domains of Sexual Functioning

Table 2 displays the ranges of correlations found between the various domains of sexual functioning, partner performance, stress and relationship satisfaction at each time point for assessment point 1 to 6. Results of our inter-correlation analysis to investigate the relationship between the different domains indicated significant associations between all domains of women’s sexual functioning, with the highest correlation consistently found (i.e. across all measurement points) between desire and arousal (r = .81 to .88; p < 0.001). In general, lubrication correlated less strongly with all other domains of women’s sexual functioning. Stress was significantly associated with all domains of women’s sexual functioning, with these associations being highest for sexual satisfaction, desire and arousal (r = -.22 to -.45, r = -.28 to -.35, and r = -.16 to -.39, and lowest for lubrication and sexual pain (r = -.11 to -.27 and r = -.02 to -.30; Table 2). No consistently significant relationship between stress and relationship satisfaction or stress and partner performance could be detected (Table 2).

**Table 2:** Cross-trait correlations range (T1-T6, n = 56–206) for the six domains of sexual function—desire, arousal, lubrication, orgasm, satisfaction, pain—and partner performance, stress and relationship satisfaction.

	Desire	Arousal	Lubrication	Pain	Orgasm	Sexual satisfaction	Partner sexual problems	Relationship satisfaction	Stress
Desire	—								
Arousal	.81**-.88**								
Lubrication	.23**-.33**	.16**-.42							
Pain	.44**-.58**	.41**-.62**	.26*-.60**						
Orgasm	.40**-.66**	.73**-.52**	.17**-.45	.36**-.58**					
Sexual satisfaction	.60**-.84**	.64**-.84**	.16**-.38	.42**-.60**	.43**-.53**				
Partner sexual problems	-.57**—.27**	-.64**—.36**	-.11—.22*	-.33*—.49**	-.56**—.38**	-.47**—.22*			
Relationship satisfaction	.24*-.55**	.18-.53**	-.18-.12	.22**-.42*	.19-.54^**^	.25^**-^.45^**^	-.34**—.07		
Stress	-.38**—.25*	-.39**—.16	-.27—.11*	-.02—.30*	-.37^**—^.12	-.45^**—^.22^*^	.03-.26*	-.21-.08	-

Note: Domains of women’s sexual functioning are inversely scored, i.e. the higher the score, the less problems are reported.

* p<0.05.

** p< 0.001.

### Within and Between-Person Variation

In Tables 3–5, linear mixed models were used to find the effect of orgasm, sexual satisfaction and erectile function on relationship satisfaction by adjusting the effect of age in separate models. A random effect was also considered for each of those variables in addition to the random intercept for the effect of each woman. As an example, the equation for the effect of orgasm on relationship satisfaction in a two level model is as follows:

$$\begin{array}{l} {\text{Relationship Satisfaction}}_{\text{ij}}\text{ = }\beta_{\text{0j}}+\beta_{1}Age_{j}+\beta_{2j}Orgasm_{ij}+e_{ij}\\ \beta_{0j}=\beta_{0}+u_{0j}\\ \beta_{2j}=\beta_{2}+u_{2j}\\ \left[\begin{array}{l} u_{0j}\\ u_{2j} \end{array}\right]\approx N(0,\Omega_{u}):\Omega_{u}=[\begin{array}{l} \sigma_{u0}^{2}\\ \sigma_{u02} \end{array}\begin{array}{l} \sigma_{u2}^{2} \end{array}]\\ e_{ij}\approx N(0,\sigma_{e}^{2}) \end{array}$$

**Table 3:** Two level random coefficient model to estimatethe effects of orgasm on relationship satisfaction.

**Fixed effects**	**Beta (SE)**	**P value**
Constant	3.14 (0.66)	<0.001
Age	-0.01 (0.01)	0.65
Orgasm	0.13 (0.07)	0.07

Random effects	Variance	P value
Woman (Intercept)	2.57 (0.50)	<0.001
Orgasm (Intercept)	0.31 (0.07)	<0.001
Orgasm and woman (Intercept)	-0.76 (0.18)	<0.001

**Table 4:** Two level random coefficient model to estimate the effects of sexual satisfaction on relationship satisfaction.

**Fixed effects**	**Beta (SE)**	**P value**
Constant	3.29 (0.65)	<0.001
Age	-0.01 (0.01)	0.59
Sexual satisfaction	0.09 (0.06)	0.17

**Random effects**	**Variance**	**P value**
Woman (Intercept)	2.34 (0.39)	<0.001
Sexual satisfaction (Intercept)	0.27 (0.05)	<0.001
Sexual satisfaction and woman (Intercept)	-0.65 (0.14)	<0.001

**Table 5:** Two level random coefficient model to estimate the effect of erectile dysfunction on relationship satisfaction.

**Fixed effects**	**Beta (SE)**	**P value**
Constant	3.96 (0.60)	0.00
Age	-0.01 (0.01)	0.36
Erectile dysfunction	-0.10 (0.08)	0.18
**Random effects**	Variance	P value
Woman (Intercept)	2.74 (0.47)	0.00
Erectile dysfunction (Intercept)	0.42 (0.09)	0.00
Erectile dysfunction and woman (Intercept)	-0.93 (0.21)	0.00

In this equation i and j indices denote the time and woman, *u*_*0j*_ is random intercept and *u*_*2j*_

is random coefficient for the effect of orgasm on relationship satisfaction and is variance-covariance matrix. This equation corresponds to the results in Table 3.

The linear regression two-level model revealed a marginally significant link between orgasm and relationship satisfaction (Beta = 0.125, P = 0.074) with this link varying significantly between women (variance = 0.308, P<0.001; Table 3). A significant negative correlation between the two random effects could be detected (r = -0.826, P<0.001), indicating a weaker association between orgasm and relationship satisfaction in women with higher orgasm baseline values (Table 3). No significant association between sexual satisfaction and partner`s sexual functioning and relationship satisfaction could be detected (P>0.150). However, similar to orgasm, the effect of these two variables varied significantly between subjects (P<0.001) and significant negative correlations between two random effects could be observed, again indicating that women with lower baseline values in the independent variables also showed weaker associations between sexual satisfaction, partner`s sexual functioning on relationship satisfaction (r<-0.80, P<0.001) (Table 4 and 5). Overall, women differed significantly from each other in terms of reported relationship satisfaction and additionally showed considerable intra-individual variation during the course of the six weeks.

Mixed linear two-level model analyses were performed to examine the extent to which overall levels of each domain of sexual functioning and contextual/interpersonal variables (e.g., desire, arousal, lubrication, orgasm, pain, sexual and relationship satisfaction, stress, partner’s sexual functioning) were related between persons (i.e., correlations among the random intercepts), and the extent to which repeatedly measured variables correlated within-persons, across time (i.e., correlations among the time-specific residuals). These associations were considered in a multivariate multilevel linear model.

Results of multivariate multilevel linear models are shown in Table 6. These multivariate models revealed that stress was significantly negatively associated with all domains of women’s sexual functioning (p<0.001). In this multivariate multilevel model, the effect of stress was adjusted for the effects of orgasm, sexual satisfaction, erectile dysfunction and relationship satisfaction. Women not only reported differing levels of stress over the course of the assessment period, but further differed from each other in how much stress they experienced and how much this influenced their sexual response. Orgasm and sexual satisfaction were both associated with all other domains of sexual function (P<0.001). Similarly, a significant link between partner performance (ED) and all domains of women`s sexual functioning (P<0.001). Except for lubrication (P = 0.717), relationship satisfaction had a significant effect on all domains of the sexual response (P<0.001). In other words, relationship satisfaction immediately preceding sexual activity was significantly related to levels of sexual functioning on all domains except for lubrication (Table 6). Inter- and intra-individual variations of all models were significant, in other word all responses varied significantly from time to time and from one woman to another.

**Table 6:** Associations between the independent variables and the six domains of female sexual functioning using mutivariate mutilevel linear modelling.

	**Desire**		**Arousal**		**Lubrication**		**Orgasm**		**Sexual Satisfaction**		**Pain**	
	**Beta (SE)**	**P value**	**Beta (SE)**	**P value**	**Beta (SE)**	**P value**	**Beta (SE)**	**P value**	**Beta (SE)**	**P value**	**Beta (SE)**	**P value**
**Stress**	-0.313 (0.043)	<0.001	-0.314 (0.048)	<0.001	-0.162 (0.051)	0.001	-0.283 (0.043)	<0.001	-0.352 (0.045)	<0.001	0.194 (0.049)	<0.001
**Orgasm**	0.613 (0.036)	<0.001	0.665 (0.039)	<0.001	0.306 (0.047)	<0.001	—	—	0.665 (0.037)	<0.001	-0.401(0.043)	<0.001
**Sexual satisfaction**	0.812 (0.030)	<0.001	0.950 (0.029)	<0.001	0.503 (0.051)	<0.001	0.562 (0.028)	<0.001	—	—	-0.653 (0.043)	<0.001
**Erectile dysfunction**	-0.208 (0.040)	<0.001	-0.399 (0.044)	<0.001	-0.318 (0.041)	<0.001	-0.168 (0.039)	<0.001	-0.345 (0.043)	<0.001	0.268 (0.040)	<0.001
**Relationship satisfaction**	0.225 (0.051)	<0.001	0.204 (0.057)	<0.001	0.021 (0.058)	0.717	0.184 (0.050)	<0.001	0.264 (0.051)	<0.001	-0.123 (0.057)	0.031

## Discussion

The present study used repeated sampling of real time data over a six week time span to investigate short-term variations in women’s sexual functioning and sensitivity to a range of psychosocial and environmental influences. To the best of our knowledge this is the first study using such an approach to help gain more in depth knowledge regarding stability and variability of women’s sexual functioning, how such fluctuations are influenced by stress, relationship satisfaction and partner performance.

### Stress and women’s sexual functioning

Whilst the effects of chronic stress are relatively well studied, exploring how female sexual response is impacted by stress on a day to day basis remains relatively under-research [32–34]. In the present study women’s subjective stress levels were assessed immediately before engaging in sexual activities, therefore representing a sensitive and somewhat broader concept of stress, which not only captures enduring stressors but also the more transient ones. A clear picture emerged in that with increasing levels of stress, women also reported lower desire, less genital and subjective arousal (consequently more pain), fewer orgasm and less sexual satisfaction. While women themselves not only reported differing levels of stress over the course of the assessment period, they further differed from each other in how much stress they experienced and how much this influenced their sexual response. In other words, comparable levels of stress were not associated with a comparable degree of impairment in sexual functioning for every woman. Such inter-individual differences in response to stress can be explained by different, more or less adaptive coping strategies and personal resource factors such as perceived self-efficacy, optimistic self-beliefs, health, optimism, and most notably also the quality of dyadic coping, attachment style, and the support from the husband [33–37].

While the woman’s subjectively felt that stress did affect her own sexual functioning it was not linked to her partner’s performance. It seems that women’s stress does not transfer to the partners and does not represent a source of stress for them. It is, however, equally possible that in a Muslim country with more patriarchal ethics women are less encouraged to talk about their problems or complain about stress, which does not foster empathic responses or sharing in the man. This is somewhat supported by the finding that stress and relationship satisfaction were independent from each other across all the assessment points. This contracts previous study findings highlighting the influence of stress (whether chronic or not) on marital quality and satisfaction [35]. Again, this might be explained by cultural differences where the woman’s stress and problems are left “outside” the marriage and considered something personal which has to be dealt with individually and therefore does not lead to alienation.

### Relationship satisfaction and women’s sexual functioning

In our study, except for lubrication, relationship satisfaction immediately preceding sexual activity was significantly related to levels of sexual functioning on all other domains of sexual response. Again, women differed significantly from each other in terms of reported relationship satisfaction and additionally showed considerable intra-individual variation during the course of the six weeks.

Our findings extend previous literature by highlighting the strong link between relationship satisfaction and sexual functioning. Numerous studies have suggested relationship dissatisfaction to be a potential causative factor in the development of FSD symptoms e.g., [22,38]. A recent study conducted by Burri and Spector, for example, indicated that relationship dissatisfaction was not only important in the pathogenesis for sexual problems experienced in the past four weeks but also for the maintenance of more enduring sexual problems [22]. Similarly, a study conducted on identical twins discordant for sexual problems (a design that allows maximization of internal validity) also found piercing evidence for the significant role of adverse relationship and interpersonal variables in the development of female sexual problems [39].

While the overall picture reveals a clear association between relationship quality and satisfaction and sexual satisfaction, it does not allow any strong assumptions regarding the direction of causality between the two phenomena, as the majority of studies used a cross-sectional design. Contrary to these studies, our design allows for more in depth exploration of this link, especially in terms of temporal precedence. Nevertheless, the influence of other factors common to relationship satisfaction and sexual functioning cannot be ruled out, hence, without further examination in a experimental design, causality cannot be assumed.

In this study, we further established a predictive effect of sexual satisfaction and partner sexual performance on women’s relationship satisfaction, and of orgasm quality. In other words, women who were sexually satisfied and reported more frequent orgasms during the last sexual activity, as well as women reporting better partner performance, were also more satisfied with their relationship. Interesting, this link was weaker in women reporting higher baseline values (more sexual satisfaction, more orgasms and better partner performance). Sexuality is an integral part of a romantic relationship and as such, sexual satisfaction and relationship happiness are both intermingled and inter-correlated to the extent that they cannot be viewed or analyzed separately from each other within a dyadic framework.

Interestingly, orgasm had an influence on subsequent relationship satisfaction. This somewhat underlines the pair-bonding theory in the heated debate surrounding the function of women’s orgasm, stating that female orgasm bonds partners, therefore not only ensuring two parents for the offspring but also making it more likely for partners to repetitively engage in sexual activities and consequently increasing the likelihood of getting pregnant [40].

### Inter- versus intra-individual variation in sexual response

Similar to reported stress levels and relationship satisfaction, women also showed considerable inter- and intra-individual variation in every stage of their sexual response. In other words, women differed significantly from each other in terms sexual functioning but also showed considerable variation in their own sexual response across the six weeks.

The findings mean that it needs to be taken into account that innate, more fundamental levels of sexual functioning exist that are relatively stable over time and that are manifested as inter-individual differences. Such inter-individual differences are likely to be influenced by different factors—most likely biologic ones—compared to the ones causing intra-individual fluctuations and phasic changes in a woman’s sexual functioning [22]. This is especially important in view of research investigating the aetiological mechanisms underlying FSD, where study designs need to consider such phenotypic differences and where the traits of interest should be carefully defined.

### Limitations

The present findings should be considered in light of several methodological limitations. The generalizability of our results may be limited, as a convenience sample of Iranian volunteers, instead of a complete random sample of the general population, was used. Most importantly, cultural factors need to be respected when interpreting and extrapolating the results. Sex and sexuality is a taboo subject in many Muslim societies and often strictly regulated. Whilst masculine sexual experiences are affirmed, women’s sexuality is limited to monogamous heterosexual marriage, where sexual purity is preserved and the woman controlled by the man. This sets a contrast to many other Western societies, where woman do enjoy more liberal and equal forms of cohabitation. Also, given the sensitive nature of the topic, women volunteering to participate might be somewhat over-represented in terms of sexual liberalism for a Muslim sample.

Further important to mention is that our study sample consisted of peri-menopausal or postmenopausal women. For this reason, representativeness of our study might be limited to the older female population, especially when considering that sexual problems are more common in peri-menopausal and post-menopausal than in the non-climacteric period [41].

We cannot exclude the possibility that our data are affected by reporting biases given the sensitive nature of the questions. Dunne and colleagues reported that surveys of sexual behavior may overestimate sexual liberalism, activity, and dysfunction (in reporting) but that this bias usually does not seriously compromise population estimates [42].

It should be noted that we used simple and restricted measures of relationship satisfaction, partner performance and stress, instead of standardized items in most cases. Nevertheless, previous studies have shown that using simple and somewhat “limited” items lead to similar results compared to standardized and more multi-facetted ones e.g., [22].

As with all methods, EMA also has its disadvantages. It is more time consuming for the participants than meeting with a clinician at intervals and it uses self-report measures which does not allow an independent check on the veracity of the data [23,29]. Another aspect that needs to be taken into consideration is reactivity which means that the sole action of monitoring sexual functioning for assessment can affect experiences and behavior. In this case the monitoring not only serves as an assessment tool but as part of a potential treatment to change behavior.

## Conclusion

To the best of our knowledge, this is the first study to use EMA to investigate the effects of stress and relationship satisfaction on women’s sexual functioning. Overall, women differed significantly from each other in terms sexual functioning but also showed considerable variation in their own sexual response across the six weeks. We further showed how relationship satisfaction and sexual functioning are strongly intermingled and cannot be viewed independently from each other. These findings support the new group of criteria introduced in the DSM-5, called “associated features” and which include for example partner factors (e.g., partner sexual problems) and/or relationship factors (e.g., poor communication, discrepancies in desire for sexual activity). Consideration of these criteria is important and necessary for clinicians when diagnosing FSD.
